# Supplementary material for: Simulation training in pancreatico-jejunostomy using an inanimate biotissue model improves the technical skills of hepatobiliary-pancreatic surgical fellows
Source: PLoS One. 2021 Jan 13;16(1):e0244915. doi: 10.1371/journal.pone.0244915 (PMC7806142; doi:10.1371/journal.pone.0244915)
Supplement: S1 Protocol — (DOCX) [file pone.0244915.s001.docx]

**Study Protocol**

1. Title: Prospective study on the training effect on the reconstruction technique in the pancreatoduodenectomy (pancreato-jejunostomy and bilio-jejunostomy) using swine and bio-tissue.
2. Name of the research institution and name of the researcher:

Responsible researcher: Akio Saiura, Professor, Hepatobiliary-Pancreatic Surgery, Juntendo School of Medicine.

Research Coordinator:

Hiroshi Imamura, Associate Professor, Hepatobiliary-Pancreatic Surgery, Juntendo School of Medicine.

Tomoya Mizuno, Assistant Professor, Hepatobiliary-Pancreatic Surgery, Juntendo School of Medicine.

Hirofumi Ichida, Assistant Professor, Hepatobiliary-Pancreatic Surgery, Juntendo School of Medicine.

Ryuji Yoshioka, Assistant Professor, Hepatobiliary-Pancreatic Surgery, Juntendo School of Medicine.

Contact Information: Hepatobiliary-Pancreatic Surgery, Juntendo School of Medicine.

3-1-3, Hongo, Bunkyo-ku, Tokyo 113-8431, Japan

Tel: +81-3-3813-3111 ext. 70815

1. The aim of the study and its clinical importance

Pancreaticoduodenectomy (PD) is a surgical procedure performed for malignant diseases and borderline malignant diseases of the pancreatic head, distal bile duct, and papilla of Vater and is one of the most invasive procedures in the field of gastrointestinal surgery. It is an anatomically complicated surgery because it excises extensive para-duodenum region including distal stomach, extrahepatic bile duct with gallbladder, pancreatic head, and the duodenum. In addition to the difficulty of excisional procedure, the reconstruction procedures after excision are also complicated and diverse such as pancreatico-jejunostomy, bile duct jejunostomy, gastro-jejunostomy, and jejunal jejunostomy.

The most dreadful postoperative complication is pancreatic juice leakage (postoperative pancreatic fistula; POPF) from the anastomotic site of the pancreas, and its incidence is reported to be approximately 20%1. While various patient-derived factors and intraoperative factors have been reported as risk factors to POPF, various variables ​​that is related to surgical skills of a surgeon were reportedly also independent risk factors based on a study on robot-assisted PD2. Therefore, it is important to be proficient in reconstruction procedures for executing PD safely. The number of cases required for achieve learning curve for PD is reportedly 50-60 cases 3, 4, however, only 3.6% of hospitals perform PD more than 28 cases per year according to the whole country surveyed data from Japan. Thus, it is not easy for a surgeon candidate for hepatobiliary-pancreatic surgery to carry out this learning curve. Among various reconstruction procedures of PD, gastrointestinal anastomosis and gastrointestinal anastomosis are also performed in other organ surgery such as gastrectomy, and it is relatively easy to accumulate experience in actual surgery. To the contrary, pancreatico-jejunostomy and bile duct jejunostomy are rarely performed other than in PD, and it can be concluded that it is almost impossible for each surgeon to reach sufficient skill only by training in on-the-job basis.

An attention is being paid for the effectiveness of anastomosis training using Biotissue as an attracting technical training method other than actual surgery. This contention was supported by a study of Biotissue robots-assisted PD where technical skill was evaluated by Objective Structured Assessment of Technical Skill (OSATS) 6. This study reported that the OSATS score was improved by training the pancreatico-jejunostomy, bile duct jejunostomy, and gastrointestinal anastomosis in PD using biotissue7. In this study, we plan to conduct training of surgeon candidates in pancreatico-jejunostomy and bile duct jejunostomy using pig organs and biotissues and examine whether or not the virtual surgery training using Biotissue model contributes to shortening the learning curve of reconstruction procedures in actual surgery through OSTAS.

4. Research method and duration

(1) Research period: Date of Ethics Committee approval -March 31, 2020.

(2) Type and design of research

Prospective/uncontrolled trial, no invasiveness, no intervention

(3) Study outline

Under the supervision of the principal investigator (Akio Saiura), surgeon candidates will learn procedures of pancreatico-jejunostomy and bile duct jejunostomy using anastomosis procedure standardized in our department by a manual which was created by the research coordinator (Ryuji Yoshioka). Then, surgical training is performed once a month using the pancreatico-jejunostomy and bile duct jejunostomy models. Training will be videotaped and scored by two responsible doctors (Akio Saiura and Ryuji Yoshioka) using attached evaluation table. The score on the evaluation table will then be disclosed to the study participants and feedback will be given on the points to be improved. Each subject will be trained 5 times in total. Researches will analyze the transition of the score during 5 training sessions and examine the effect of the training.

Lecture by attending surgeon

Trial 1

Trial 2

Trial 3

Trial 4

Final trial

Assessment

Feedback

Assessment

Feedback

Assessment

Feedback

Assessment

Feedback

Assessment

4) Overview of study drug / medical device information

Not applicable

1. Test drug name: Product name (generic name)

Not applicable

1. Predicted adverse events (predicted defects in the case of medical devices)

None

(5) Administration method of test drug

Not applicable

(6) Regulations on concomitant medications (therapy)

Not applicable

(7) Regulations on weight loss and drug suspension

Not applicable

(8) Case registration and allocation method

Not applicable

(9) Scheduled research participation period of research subjects

Research subjects will participate in the observation period of 6 months after consent.

(10) Observation and inspection items

Evaluation table

5. Selection policy of research participants

(1) Research participants

Surgical trainees in Division of Hepatobiliary-Pancreatic Surgery, Juntendo University School of Medicine as of April 1, 2019.

(2) Selection criteria

① Surgical trainees in Division of Hepatobiliary-Pancreatic Surgery, Juntendo University School of Medicine as of April 1, 2019.

② Those who have received sufficient explanation before participating in this research, and have provided the voluntary consent of the research subject.

(3) Exclusion criteria

① Those who are judged by the principal investigator to be inappropriate as research subjects.

(4) Cancellation criteria

① When the research subject declines participation in the research or withdraws consent.

② When the entire research is canceled.

③ When the principal investigator and the research coordinator decide that it is appropriate to discontinue the research for other reasons.

6. Basis for scientific rationality of research

(1) Target number of participants and its rationale

Nine participants.

[Rationale for setting]

There are a total of 13 medical staff members in Division of Hepatobiliary-Pancreatic Surgery, Juntendo University School of Medicine as of April 1, 2019. Excluding four who are in a leadership position as responsible surgeons, remaining nine are candidates.

(2) Statistical analysis method

Nine research participants will each be trained five times in total, and the evaluator will score each procedure using the evaluation table. The transition of the score after 5 trainings is analyzed using the repeated ANOVA method. In addition, in order to examine the validity of the evaluation by the evaluator, the scores between the evaluators are analyzed using the κ test.

7. Procedures for receiving informed consent pursuant to the provisions of Article 12 of the Ethics Guidelines

The consent explanation document approved by the ethics committee of the Faculty of Medicine will be given to the research subject, sufficient written and oral explanations will be given, and the voluntary consent of the research subject will be obtained in writing. When information that affects the consent of the research subject is obtained, or when the research plan, etc. that affects the consent of the research subject is changed, the information is promptly provided to the research subject. The intention of the research subject will be confirmed in advance as to whether or not to participate in the research, and the consent explanation document will be revised with the approval of the ethics committee of the Faculty of Medicine in advance, and the re-consent of the research subject will be obtained.

The consent explanation document shall include the following contents.

① The name of the research and the permission of the director of the research institution (dean of the Faculty of Medicine) for conducting the research.

② Name of the research institution and name of the principal investigator (when conducting research jointly with other research institutes, joint research institute, it includes the name of the research institution and the name of the principal investigator of the joint research institution).

1. Purpose and significance of research.

④ Research method (including the purpose of use of samples and information obtained from the research subject) and period.

1. Reason for being selected as a research subject.
2. Burden on research subjects and expected risks and benefits.

⑦ Even if you agree that the research will be conducted or continued, you can withdraw it at any time (research subjects, etc.).

⑧ Research participants will not by to be treated unfavorably disagreeing with or withdrawing the consent to carry out or continue the research.

⑨ Method of disclosing information on research.

⑩ At the request of the research participants, they can obtain the research plan and materials related to the research method within the range that does not hinder the protection of personal information of other research subjects and the originality of the research.

⑪ Handling of personal information (including the method when anonymizing, and that when creating anonymously processed information or non-identified processed information).

⑫ How to store and dispose of samples and information.

⑬ Conflicts of interest related to research by research institutes such as funding sources for research and conflicts of interest related to research by researchers.

⑭ Response to consultations from research participants and related parties.

⑮ If there is an economic burden or reward for the research participants, that fact and its contents.

⑯ Matters related to other treatment methods in the case of research involving medical practice that exceeds normal medical care.

⑰ In the case of research involving medical practice that exceeds normal medical care, measures regarding the provision of medical care to the research subjects after the research is conducted.

⑱ In the case of research involving invasion, whether or not there is compensation for health damage caused by the research and its contents.

⑲ If there is a possibility that the samples and information obtained from the research subjects will be used for future research that will not be specified at the time of obtaining the consent of the research subjects, or will be provided to other research institutes, its content that is expected at the time of receiving consent to that effect.

⑳ In the case of research involving invasion (excluding minor invasion) and intervention, on the premise that the confidentiality of the research subject is preserved, those engaged in monitoring and auditing, as well as those engaged in auditing. The ethics committee of the Faculty of Medicine will browse samples and information about the research subjects to the extent necessary.

8. Handling of personal information, etc. (Including the method when anonymizing, and the fact when creating anonymously processed information or non-identified processed information)

When handling samples related to research implementation, the researches will manage them with a research ID that is not related to the personal information of the research subject, and give due consideration to the confidentiality protection of the research subject. The created correspondence table is stored in a locked locker in the Division of Hepatobiliary-Pancreatic Surgery Laboratory, and is strictly managed by Yuji Yoshioka. When publishing the results of a study, the researches do not include information that can identify the research subject. In addition, the samples of the research subjects obtained in the research will not be used for purposes other than the purpose of the research.

9. The burden and expected risks and benefits to the research subjects, their comprehensive evaluation, and measures to minimize the burden and risks

(1) Expected profit

By participating in this study, it is expected that the surgical technique of the research participants will be improved.

(2) Expected disadvantages (side effects)

Nothing special.

(3) Response to research subjects when adverse events occur

When an adverse event is observed, the investigators should take appropriate measures immediately and describe it in the medical record and case report form. In addition, if treatment for adverse events is required, the investigators should inform the study participants to that effect.

(4) Changes to research plans.

The investigators collect and review the information necessary to safely carry out clinical research. In addition, when new safety information is obtained, the research plan and consent explanation document will be changed as necessary. Before changing or revising the research plan or consent explanation document, the approval of the ethics committee of the Faculty of Medicine is required.

(5) Criteria for discontinuation for individual research participants

[Response when research is stopped]

If the principal investigator or the research coordinator determines that it is impossible to continue the research for each research subject for the following reasons, the research for the research subject will be discontinued. At that time, the principal investigator will explain the reason for discontinuation to the subject as necessary. In addition, regarding the treatment of the research subjects after discontinuation, we will respond in good faith so as not to disadvantage the research subjects.

[Cancellation criteria]

1. When the research subject offers to decline participation in the research or withdraws consent.
2. When the entire research is canceled.

③ When the person in charge of research determines that it is appropriate to discontinue the research for other reasons.

10. Method of storing and disposing of samples and information (including materials related to information used in research)

The principal investigator is responsible for important documents related to the implementation of research (copy of application documents, notification document from the Dean of the Faculty of Medicine, copy of various applications/reports, consent form, copy of case report, and trust in other data. Documents or records necessary for guaranteeing reliability) should be stored in accordance with the "Standard Business Procedures for Storage of Samples and Information Related to Medical Research for Humans" after the research is stopped or completed. They will be stored in a locked locker in the Division of Hepatobiliary-Pancreatic Surgery Office until the day after 5 years, after which it should be carefully discarded.

11. Report contents and method to the director of the research institution (department of medicine)

The report to the director of the research institution (dean of the Faculty of Medicine) is as follows:

(1) Once a year, the status of research implementation will be reported in Form No. 8, and the suitability of continuing research will be examined by the Ethics Committee of the Faculty of Medicine.

(2) If the documents used for the examination at the time of application are changed, the principal investigator applies to the Dean of the School of Medicine in advance and obtain the approval of the Ethics Committee of the School of Medicine in advance.

(3) If a serious adverse event occurs in the hospital, promptly report it to the Dean of the School of Medicine using Form No. 6 and have the ethics committee of the School of Medicine examine the suitability of continuing the research.

(4) When important information on the efficacy and safety of the study drug, etc. is obtained, the opinion of the principal investigator is stated in Form No. 7 and reported to the Dean of the Faculty of Medicine, and the Faculty of Medicine regarding the appropriateness of continuing the research. It will be examined by the Ethics Committee.

(5) At the end of the study (including the case of discontinuation or interruption), the principal investigator will report to the Dean of the Faculty of Medicine in Form No. 9.

12. Conflicts of interest related to research by research institutes, such as funding sources for research, and conflicts of interest related to research by researchers

This research will be carried out by receiving materials of less than 2 million yen from Johnson & Johnson Co., Ltd. However, Johnson & Johnson Co., Ltd. is not involved in the implementation, analysis, or reporting of research, and the research results are not distorted in favor of Johnson & Johnson Co., Ltd..

In addition, the researchers of this research are in accordance with the "Juntendo University Medical Research Conflict Management Regulations" and the "Standard Business Procedure Manual for Conflicts of Interest Related to Human Medical Research", Juntendo University School of Medicine Medical Research Benefits. The necessary items shall be reported to the Conflict Management Committee and shall be examined.

13. How to disclose information about research

This research is registered in the public database set up by the National University Hospital Directors' Meeting (UMIN). The results obtained in the above will be presented at the Gastroenterological Surgery Society by the research coordinator Yuji Yoshioka after the research is completed, and will be published as a paper in a specialized academic journal in the field of gastrointestinal surgery. In either case, only statistically processed results will be published, and no personal information that can identify the individual research subject will be published.

14. Responding to consultations, etc. from research subjects and related parties

For consultations from research subjects and related parties, please contact the following consultation desk:

[Consultation counter]

Research coordinator: Ryuji Yoshioka, Associate Professor, Division of Hepatobiliary-Pancreatic Surgery.

Hepatobiliary-Pancreatic Surgery, 3-1-3 Hongo, Bunkyo-ku, Tokyo 113-8431 Juntendo University School of Medicine

+81-3-3813-3111 (ext. 70815)

15.If there is an economic burden or reward for the research participants, etc., that fact and its contents

Since the materials used in this research are provided free of charge by Johnson & Johnson Co., Ltd., there is no cost burden on the research subjects by participating in the research.

16. In the case of invasive research, response to a serious adverse event

If a serious adverse event occurs, the principal investigator will take necessary measures and report it to the Dean of the Faculty of Medicine using Form No. 6, and the researchers involved in the implementation of the research (multicenter research). In this case, the information on the adverse event will be shared with the principal investigator of the other facility. In addition, regarding the reporting procedure, the principal investigator will follow the "Standard Business Procedure Manual for Reporting and Response to Serious Adverse Events and Problems Related to Medical Research for Humans".

A serious adverse event or malfunction is one that falls under any of the following:

1. Events that lead to death.
2. Life-threatening event.
3. Those that require hospitalization for treatment or extension of hospitalization period.
4. Permanent or significant failure/dysfunction.
5. Those that cause birth defects in offspring.

17. In the case of invasive research, the content of compensation for health damage caused by the research

There is no compensation in the unlikely event that the research subject suffers a health hazard due to participation in this research. If a participant suffers from health problems, she/she will be taken appropriate measures and be taken necessary measures such as examinations and treatments within the subject's insurance medical treatment.

18. In the case of research that involves medical practice that exceeds normal medical care, measures regarding the provision of medical care to the research subjects after the research is conducted.

Not applicable.

19. If there is a possibility that important findings regarding the health of the research subject, genetic characteristics that can be inherited by offspring can be obtained as a result of conducting the research, the research results (including accidental findings) of the research subject are included)

There is no possibility that important clinical trials regarding the health and genetic characteristics of the study subjects will be obtained as the study is conducted.

20. When outsourcing a part of research-related work, the content of the work and the supervision method of the outsourced person

We do not outsource any part of the research work.

21. If there is a possibility that the samples/information obtained from the research participants will be used for future research that is not specified at the time of obtaining the consent of the research subject or will be provided to other research institutes What is expected at the time of receiving consent to that effect?

The materials and information obtained in this research are not specified at the time of obtaining consent from the research participants. There is no possibility that they will be used for future research or provided to other research institutes.

22. When conducting monitoring and audits pursuant to the provisions of Code of Ethics No. 21

Since this is a non-invasive study, monitoring and auditing will not be conducted.
