# Supplementary material for: Simulation training in pancreatico-jejunostomy using an inanimate biotissue model improves the technical skills of hepatobiliary-pancreatic surgical fellows
Source: PLoS One. 2021 Jan 13;16(1):e0244915. doi: 10.1371/journal.pone.0244915 (PMC7806142; doi:10.1371/journal.pone.0244915)
Supplement: S2 Data — (DOCX) [file pone.0244915.s003.docx]

# **研　究　計　画　書**

## **1．研究の名称**

　膵頭十二指腸切除術の再建手技（膵空腸吻合および胆管空腸吻合）におけるブタ臓器およびbiotissueによる膵空腸・胆管空腸吻合モデルを用いたトレーニングの及ぼす効果についての前向き非対照試験

## **2．研究の実施体制（研究機関の名称及び研究者等の氏名を含む。）**

【研究責任者】

順天堂大学医学部附属順天堂医院肝胆膵外科・教授　　　氏名　齋浦明夫

【研究分担者】

順天堂大学医学部附属順天堂医院肝胆膵外科・准教授　　氏名　今村宏

順天堂大学医学部附属順天堂医院肝胆膵外科・准教授　　氏名　三瀬祥弘

順天堂大学医学部附属順天堂医院肝胆膵外科・助教　　　氏名　水野智哉

順天堂大学医学部附属順天堂医院肝胆膵外科・助教　　　氏名　市田洋文

順天堂大学医学部附属順天堂医院肝胆膵外科・助教　　　氏名　吉岡龍二

【連絡先】

〒113-8431東京都文京区本郷3-1-3　　順天堂大学医学部附属順天堂医院

肝胆膵外科　　　　03-3813-3111（内線70815）

## **3．研究の目的及び意義**

膵頭十二指腸切除術（Pancreaticoduodenectomy; PD）は膵頭部・遠位胆管・十二指腸乳頭部の悪性疾患や境界悪性疾患に対して施行される術式であり、消化器外科領域では最も侵襲の大きな手術の一つである。解剖学的に複雑な傍十二指腸領域の切除であり、切除臓器も胃の遠位、胆嚢を含む肝外胆管、膵頭部、十二指腸が含まれ、切除の難しさのみならず、切除後の再建手技も代表的なものとして膵空腸吻合・胆管空腸吻合、胃空腸吻合、そして空腸空腸吻合と複雑かつ多彩である。

術後合併症として最も重要なものが膵空腸吻合部からの膵液漏（Postoperative pancreatic fistula; POPF）であり、その発生率は20％程度と報告されている[^1^](#_ENREF_1)。POPFのリスク因子として様々な患者側因子・術中因子が報告されている一方で、ロボット支援下PDについての研究ではあるが、外科医の手術技量を評価した数値が患者側因子とは独立してPOPFの予測因子となることが報告されており[^2^](#_ENREF_2)、正確な再建手技に習熟することはPDを安全に施行するうえで重要である。しかしPDのラーニングカーブに必要な症例数は50-60例とされており[^3^](#_ENREF_3)^,^ [^4^](#_ENREF_4)、日本のDPCデータを用いた論文では年間28例を超えるPDを施行している病院は全体の3.6％程度[^5^](#_ENREF_5)であるため、肝胆膵外科医を志す外科医がこのラーニングカーブを超えることは容易ではない。PD切除後の再建手技のうち、胃空腸吻合および空腸空腸吻合の手技については胃切除などの他臓器手術でも施行され、実際の手術での経験を積み重ねることは比較的容易であるが、膵空腸吻合と胆管空腸吻合についてはPD以外の手術で施行することはまれであり、実地診療での修練のみですべての外科医が十分な技量に到達することはほぼ不可能といえる。

実際の手術以外での技術的訓練としてBiotissueを用いた吻合訓練の有効性が注目されており、Objective Structured Assessment of Technical Skill(以下OSATS)[^6^](#_ENREF_6)という外科的手技の技術評価指標を用いてロボット支援下PDにおける膵空腸吻合・胆管空腸吻合・胃空腸吻合をbiotissueを用いて訓練を行いOSATSスコアの改善を認めたとする報告がある[^7^](#_ENREF_7)。今回我々は当教室医局員のPD技術教育としてブタ臓器およびbiotissueを用いた膵空腸吻合・胆管空腸吻合モデルを用いてトレーニングを行い、客観的評価指標を用いて技量の定量的評価の推移を行い、生体外トレーニングが実際の手術において再建手技のラーニングカーブ短縮に寄与するかを検討する。

## **4．研究の方法及び期間**

（1）研究実施期間：倫理委員会承認日～西暦2021年3月31日

（2）研究の種類・デザイン

前向き・非対照試験、侵襲なし、介入なし

（3）試験のアウトライン

研究責任者（齋浦明夫）監修のもと、研究分担者（吉岡龍二）が作成した吻合手技マニュアルを用いて、当科で標準化して行っている胆管空腸吻合および膵空腸吻合の手技について座学で学ぶ。1か月に一度、胆管空腸吻合及び膵空腸吻合モデルを用いて実技トレーニングを行う。実技トレーニングはビデオ撮影し、2名の責任医師（齋浦明夫・吉岡龍二）が添付の評価表を用いて採点する。評価表のスコアはその後研究対象者に開示し、改善すべき点についてフィードバックを行う。被験者は各自計5回のトレーニングを行う。5回のトレーニング時のスコアの推移を解析し、トレーニングの効果を検討する。


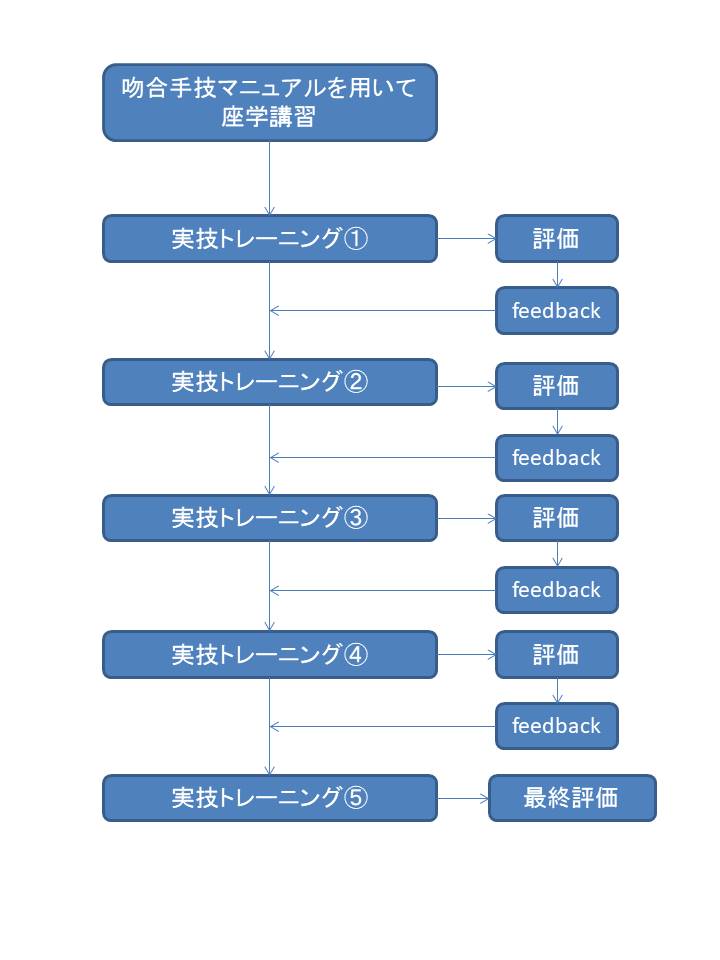


　 （4）試験薬の概要／医療機器の情報

　　　該当なし

①試験薬名：商品名（一般名）

該当なし

　②予測される有害事象（医療機器の場合は予測される不具合）

　 特に無し

（5）試験薬の投与方法

該当なし

（6）併用薬（療法）についての規定

該当なし

（7）減量および休薬のついての規定

該当なし

（8）症例登録、割付の方法

該当なし

（9）研究対象者の研究参加予定期間

研究対象者は同意後、6ヶ月の観察期間で参加する

(10) 観察および検査項目

①評価表

【観察および検査スケジュール表】

## **5．研究対象者の選定方針**

（1）研究対象者

順天堂大学医学部附属順天堂医院　肝胆膵外科に西暦2019年4月1日の時点で在籍する医局員を対象とする。

（2）選択基準

①順天堂大学医学部附属順天堂医院　肝胆膵外科に西暦2019年4月1日の時点で在籍する医局員

②本研究への参加にあたり十分な説明を受けた後、十分な理解の上、研究対象者本人の自由意思による文書同意が得られた方

（3）除外基準

①その他、研究責任者が研究対象者として不適当と判断した方

上記（1）研究対象者のうち、（2）選択基準をすべて満たし、かつ（3）除外基準のいずれにも該当しない場合を適格とする。

（4）中止基準

①　研究対象者から研究参加の辞退の申し出や同意の撤回があった場合

②　本研究全体が中止された場合

③　その他の理由により、研究責任者および研究分担者が研究の中止が適当と判断した場合

## **6．研究の科学的合理性の根拠**

（1）目標症例数とその設定根拠

9例

【設定根拠】

順天堂大学医学部附属順天堂医院　肝胆膵外科に西暦2019年4月1日の時点で在籍する医局員が計13名、そのうち責任術者として指導的立場にある4名を除く。

（2）統計解析方法

　9名の研究対象者は各自計5回のトレーニングを行い、それぞれの手技について評価者が評価表を用いてスコアをつける。5回のトレーニングによるスコアの推移をrepeated ANOVA法を用いて解析する。また評価者による評価の妥当性を検討するため、評価者間のスコアをκ検定を用いて解析する。

## **7．倫理指針第 12 の規定によるインフォームド・コンセントを受ける手続等**（インフォーム ド・コンセントを受ける場合には、同規定による説明及び同意に関する事項を含む。）

医学部倫理委員会で承認の得られた同意説明文書を研究対象者に渡し、文書および口頭による十分な説明を行い、研究対象者の自由意思による同意を文書　で取得する。研究対象者の同意に影響を及ぼす情報が得られたときや、研究対象者の同意に影響を及ぼすような研究計画書等の変更が行われるときは、速やかに研究対象者に情報提供し、研究に参加するか否かについて研究対象者の意思を予め確認するとともに、事前に医学部倫理委員会の承認を得て同意説明文書等の改訂を行い、研究対象者の再同意を得ることとする。

同意説明文書には、以下の内容を含むものとする。

　　　　　①研究の名称及び当該研究の実施について研究機関の長（医学部長）の許可を受けている旨

　　　　　②研究機関の名称及び研究責任者の氏名（他の研究機関と共同して研究を実施する場合には、共同研

究機関の名称及び共同研究機関の研究責任者の氏名を含む。）

　　　　　③研究の目的及び意義

　　　　　④研究の方法（研究対象者から取得された試料・情報の利用目的を含む。）及び期間

　　　　　⑤研究対象者として選定された理由

　　　　　⑥研究対象者に生じる負担並びに予測されるリスク及び利益

　　　　　⑦研究が実施又は継続されることに同意した場合であっても随時これを撤回できる旨（研究対象者等

からの撤回の内容に従った措置を講じることが困難となる場合があるときは、その旨及びその理由）

　　⑧研究が実施又は継続されることに同意しないこと又は同意を撤回することによって研究対象者等が

不利益な扱いを受けない旨

　　　　　⑨研究に関する情報公開の方法

　　　　　⑩研究対象者等の求めに応じて、他の研究対象者等の個人情報等の保護及び当該研究の独創性の確保に支障がない範囲内で研究計画書及び研究の方法に関する資料を入手又は閲覧できる旨並びにその入手又は閲覧の方法

　　　　　⑪個人情報等の取扱い（匿名化する場合にはその方法、匿名加工情報又は非識別加工情報を作成する場合にはその旨を含む。）

　　　　　⑫試料・情報の保管及び廃棄の方法

　　　　　⑬研究の資金源等、研究機関の研究に係る利益相反及び個人の収益等、研究者等の研究に係る利益相反に関する状況

　　　　　⑭研究対象者等及びその関係者からの相談等への対応

　　　　　⑮研究対象者等に経済的負担又は謝礼がある場合には、その旨及びその内容

　　　　　⑯通常の診療を超える医療行為を伴う研究の場合には、他の治療方法等に関する事項

　　　　　⑰通常の診療を超える医療行為を伴う研究の場合には、研究対象者への研究実施後における医療の提供に関する対応

　　　　　⑱侵襲を伴う研究の場合には、当該研究によって生じた健康被害に関する補償の有無及びその内容

　　　　　⑲研究対象者から取得された試料・情報について、研究対象者等から同意を受ける時点では特定されない将来の研究のために用いられる可能性又は他の研究機関に提供する可能性がある場合には、その旨と同意を受ける時点において想定される内容

　⑳侵襲（軽微な侵襲を除く。）を伴う研究であって介入を行うものの場合には、研究対象者の秘密が保全されることを前提として、モニタリングに従事する者及び監査に従事する者並びに医学部倫理委員会が、必要な範囲内において当該研究対象者に関する試料・情報を閲覧する旨

## **8．個人情報等の取扱い**（匿名化する場合にはその方法、匿名加工情報又は非識別加工情報を作成する場合にはその旨を含む。）

研究実施に係る試料等を取扱う際は、研究対象者の個人情報とは関係ない研究用ＩＤを付して管理し、研究対象者の秘密保護に十分配慮する。作成した対応表は肝胆膵外科研究室の鍵のかかるロッカーで保管され、吉岡龍二が厳重に管理する。研究の結果を公表する際は、研究対象者を特定できる情報を含まないようにする。また、研究の目的以外に、研究で得られた研究対象者の試料等を使用しない。

## **9．研究対象者に生じる負担並びに予測されるリスク及び利益、これらの総合的評価並びに当該負担及びリスクを最小化する対策**

（1）予想される利益

本研究へ参加することによって研究対象者の手術手技向上が期待できる。

（2）予想される不利益（副作用）

特になし

　 （3）有害事象発生時の研究対象者への対応

研究担当者は、有害事象を認めたときは直ちに適切な処置を行うとともに、診療録ならびに症例報告書に記載する。また、有害事象に対する治療が必要となった場合には、研究対象者にその旨を伝える。

（4）研究計画書等の変更

臨床研究を安全に実施する上で必要な情報を収集し、検討する。また、新たな安全性情報等が得られた場合、必要に応じて研究計画書および同意説明文書を変更する。研究計画書や同意説明文書の変更または改訂を行う場合は、あらかじめ医学部倫理委員会の承認を必要とする。

　　 （5）個々の研究対象者における中止基準

【研究中止時の対応】

研究責任者または研究分担者は、次に挙げる理由で個々の研究対象者について研究継続が不可能と判断した場合には、当該研究対象者についての研究を中止する。その際は、必要に応じて中止の理由を被験者に説明する。また、中止後の研究対象者の治療については、研究対象者の不利益とならないよう、誠意を持って対応する。

【中止基準】

①　研究対象者から研究参加の辞退の申し出や同意の撤回があった場合

②　本研究全体が中止された場合

③　その他の理由により、研究担当者が研究の中止が適当と判断した場合

## **10．試料・情報（研究に用いられる情報に係る資料を含む。）の保管及び廃棄の方法**

研究責任者は、研究等の実施に係わる重要な文書（申請書類の控え、医学部長からの通知文書、各種申請書・報告書の控、同意書、症例報告書等の控、その他データの信頼性を保証するのに必要な書類または記録等）の保管については、「人を対象とする医学系研究に係る試料及び情報等の保管に関する標準業務手順書」に従って行い、研究の中止または終了後5年が経過した日までの間、肝胆膵外科医局内の鍵のかかるロッカーにて保存し、その後は個人情報に注意して廃棄する。

## **11．研究機関の長（医学部長）への報告内容及び方法**

　研究機関の長（医学部長）への報告については下記の通りとする。

（1）年1回、研究実施状況について様式第8号により報告し、研究継続の適否について医学部倫理委員会の審査を受ける。

（2）申請時審査に用いた書類に変更が生じる場合には、事前に医学部長に申請し、あらかじめ医学部倫理委員会の承認を受ける。

（3）院内で重篤な有害事象が発生した場合は、速やかに様式第6号により医学部長に報告し、研究継続の適否について医学部倫理委員会の審査を受ける。

（4）試験薬等の有効性・安全性に関する重要な情報が得られた場合は、様式第7号により、研究責任者の見解を記載し、医学部長に報告し、研究継続の適否について医学部倫理委員会の審査を受ける。

（5）研究の終了時（中止または中断の場合を含む）には、様式第9号により、医学部長に報告する。

## **12．研究の資金源等、研究機関の研究に係る利益相反及び個人の収益等、研究者等の 研究に係る利益相反に関する状況**

本研究は、ジョンソン・エンド・ジョンソン(株)から200万円未満の資材提供を受けて実施する。しかし、ジョンソン・エンド・ジョンソン(株)は研究の実施、解析、報告に係わることはなく、研究結果がジョンソン・エンド・ジョンソン(株)に有利に歪められることはない。

また、本研究の研究者は、「順天堂大学医学系研究利益相反マネジメント規程」および「人を対象とする医学系研究に係る利益相反に関する標準業務手順書」に則り、順天堂大学医学部医学系研究利益相反マネジメント委員会に必要事項を申告し、その審査を受けるものとする。

## **13．研究に関する情報公開の方法**

　本研究は、国立大学附属病院長会議(UMIN)が設置している公開データベースに登録する。また、本研究

で得られた結果は、研究終了後に研究分担者吉岡龍二が消化器外科学会で発表し、消化器外科学領域の専門学術誌で論文として公表する予定である。いずれの場合においても公表する結果は統計的な処理を行ったものだけとし、研究対象者の個人を特定できるような個人情報は一切公表しない。

## **14．研究対象者等及びその関係者からの相談等への対応**

　　　研究対象者等及びその関係者からの相談については、下記相談窓口にて対応する。

　　　【相談窓口】

　　　　研究分担者　肝胆膵外科　氏名　吉岡龍二

〒113-8431東京都文京区本郷3-1-3　　順天堂大学医学部附属順天堂医院

肝胆膵外科　　　　03-3813-3111（内線70815）

## **15．研究対象者等に経済的負担又は謝礼がある場合には、その旨及びその内容**

本研究で用いる資材はジョンソン・エンド・ジョンソン（株）からの無償提供されるため、研究に参加することによる研究対象者の費用負担は発生しない。

## **16．侵襲を伴う研究の場合には、重篤な有害事象が発生した際の対応**

研究責任者は、重篤な有害事象（不具合）が発生した場合は、必要な処置を行うとともに様式第6号により医学部長へ報告し、当該研究の実施に携わる研究者等（多施設共研究の場合は他の施設の研究責任者）に対して、当該有害事象の情報を共有する。また、報告手順については「人を対象とする医学系研究に係る重篤な有害事象及び不具合等の報告・対応に関する標準業務手順書」に従う。

　　　重篤な有害事象又は不具合とは、以下のいずれかに該当するものをいう。

　　　　①死に至るもの

　　　　②生命を脅かすもの

　　　　③治療のための入院又は入院期間の延長が必要となるもの

　　　　④永続的又は顕著な障害・機能不全に陥るもの

　　　　⑤子孫に先天異常を来すもの

## **17．侵襲を伴う研究の場合には、当該研究によって生じた健康被害に対する補償の内容**

本研究への参加に起因して、万が一、研究対象者に健康被害が生じた場合に、補償等はない。研究対　象者に健康被害が発生した場合は、適切な処置を講じ被験者の保険診療内で検査や治療等、必要な処置を行う。

## **18．通常の診療を超える医療行為を伴う研究の場合には、研究対象者への研究実施後における医療の提供に関する対応**

該当なし

## **19．研究の実施に伴い、研究対象者の健康、子孫に受け継がれ得る遺伝的特徴等に関する重要な知見が得られる可能性がある場合には、研究対象者に係る研究結果（偶発的所見を含む。）の取扱い**

研究の実施に伴い、研究対象者の健康・遺伝的特徴等に関する重要な治験が得られる可能性は無い

## **20．研究に関する業務の一部を委託する場合には、当該業務内容及び委託先の監督方法**

研究に関する業務の一部を委託することは無い

## **21．研究対象者から取得された試料・情報について、研究対象者等から同意を受ける時点では特定されない将来の研究のために用いられる可能性又は他の研究機関に提供する可能性がある場合には、その旨と同意を受ける時点において想定される内容**

本研究で取得された資料・情報について研究対象者等から同意を受ける時点では特定されない将来の研究のために用いられる可能性やほかの研究機関に提供する可能性は無い

## **22．倫理指針第 21 の規定によるモニタリング及び監査を実施する場合には、その実施体制及び実施手順**

侵襲を伴わない非介入研究であるため、モニタリング・監査については実施しない。

## **23．参考文献リスト**

1. Sanchez-Velazquez P, Muller X, Malleo G, Park JS, Hwang HK, Napoli N et al. Benchmarks in Pancreatic Surgery: A Novel Tool for Unbiased Outcome Comparisons. Ann Surg. 2019;

2. Hogg ME, Zenati M, Novak S, Chen Y, Jun Y, Steve J et al. Grading of Surgeon Technical Performance Predicts Postoperative Pancreatic Fistula for Pancreaticoduodenectomy Independent of Patient-related Variables. Ann Surg. 2016; 264: 482-491

3. Fisher WE, Hodges SE, Wu MF, Hilsenbeck SG, Brunicardi FC. Assessment of the learning curve for pancreaticoduodenectomy. Am J Surg. 2012; 203: 684-690

4. Schmidt CM, Turrini O, Parikh P, House MG, Zyromski NJ, Nakeeb A et al. Effect of hospital volume, surgeon experience, and surgeon volume on patient outcomes after pancreaticoduodenectomy: a single-institution experience. Arch Surg. 2010; 145: 634-640

5. Yoshioka R, Yasunaga H, Hasegawa K, Horiguchi H, Fushimi K, Aoki T et al. Impact of hospital volume on hospital mortality, length of stay and total costs after pancreaticoduodenectomy. Br J Surg. 2014; 101: 523-529

6. Martin JA, Regehr G, Reznick R, MacRae H, Murnaghan J, Hutchison C et al. Objective structured assessment of technical skill (OSATS) for surgical residents. Br J Surg. 1997; 84: 273-278

7. Tam V, Zenati M, Novak S, Chen Y, Zureikat AH, Zeh HJ, 3rd et al. Robotic Pancreatoduodenectomy Biotissue Curriculum has Validity and Improves Technical Performance for Surgical Oncology Fellows. J Surg Educ. 2017; 74: 1057-1065
