# Supplementary material for: Simulation training in pancreatico-jejunostomy using an inanimate biotissue model improves the technical skills of hepatobiliary-pancreatic surgical fellows
Source: PLoS One. 2021 Jan 13;16(1):e0244915. doi: 10.1371/journal.pone.0244915 (PMC7806142; doi:10.1371/journal.pone.0244915)
Supplement: Attachment — Submitted filename: Response to Reviewers.docx [file pone.0244915.s004.docx]

Dr. Robert Jeenchen Chen,

Academic Editor of PLOS ONE

We would like to thank you and all the reviewers of our manuscript titled“Simulation training in pancreatico-jejunostomy using an inanimate biotissue model improves the technical skills of hepatobiliary-pancreatic surgical fellows”. for their insightful comments. Their suggestions have substantially enhanced the piece. We have understood each of their comments, directly addressed them as delineated below, and revised the manuscript accordingly. We thank you and the reviewers for your efforts toward bringing our novel findings to press.

Sincerely yours,

Akio Saiura M.D., Ph.D.

Department of Hepatobiliary-Pancreatic Surgery, Juntendo University Graduate School of Medicine

2-1-1 Hongo, Bunkyo-ku, Tokyo 113-8421, Japan

Tel: +81-3-3813-3111

Fax: +81-3-3818-7589

e-mail: a-saiura@juntendo.ac.jp

Journal Requirements:

When submitting your revision, we need you to address these additional requirements.

1) Please ensure that your manuscript meets PLOS ONE's style requirements, including those for file naming. The PLOS ONE style templates can be found at

<https://journals.plos.org/plosone/s/file?id=wjVg/PLOSOne_formatting_sample_main_body.pdf> and

<https://journals.plos.org/plosone/s/file?id=ba62/PLOSOne_formatting_sample_title_authors_affiliations.pdf>

**Response:** We revised the manuscript in accordance with PLOS ONE’s style

2) We note that you have stated that you will provide repository information for your data at acceptance. Should your manuscript be accepted for publication, we will hold it until you provide the relevant accession numbers or DOIs necessary to access your data. If you wish to make changes to your Data Availability statement, please describe these changes in your cover letter and we will update your Data Availability statement to reflect the information you provide.

**Response:** We did not change Data Availability statement in the revised manuscript.

3) Please include captions for your Supporting Information files at the end of your manuscript, and update any in-text citations to match accordingly. Please see our Supporting Information guidelines for more information: <http://journals.plos.org/plosone/s/supporting-information>.

**Response:** A caption for Supporting Information was added in the revised manuscript.

4)  In your Methods section, please provide additional information about the participant recruitment method and the demographic details of your participants. Please ensure you have provided sufficient details to replicate the analyses such as: a) the recruitment date range (month and year), b) a description of any inclusion/exclusion criteria that were applied to participant recruitment, c) a table of relevant demographic details, d) a description of how participants were recruited, and e) descriptions of where participants were recruited and where the research took place.

**Response:** According to the editor’s comments, we added the following sentences in the Method section in the revised manuscript:

Page 5, line 102-104 (new):

The biotissue PJ drills were conducted once a month between September 2019 and January 2020 at the Department of Hepatobiliary-Pancreatic Surgery, Juntendo University Hospital.

Page 5, line 111-112, Page 6 line 113-116 (new):

**Participants**

Inclusion criterion of the present study was as follows: surgical fellows who were affiliated with the Department of Hepatobiliary-Pancreatic Surgery, Juntendo University Hospital as of September 2019. Because all of eight affiliated fellows, thus, candidates, participated in the study from the educational aspect, sample size was not calculated.

5) Please provide a sample size and power calculation in the Methods, or discuss the reasons for not performing one before study initiation.

**Response:** Please see the reply to comment 4)

6) During our internal checks, we noted that your study does not fit the WHO definition of a clinical trial. Therefore, the TREND checklist is not appropriate for this trial design and we suggest removing it from the supplementary data.

**Response:** According to the editor’s comments, we removed the TREND checklist from the revised manuscript.

[Note: HTML markup is below. Please do not edit.]

Reviewers' comments:

Reviewer's Responses to Questions

**Comments to the Author**

1. Is the manuscript technically sound, and do the data support the conclusions?

The manuscript must describe a technically sound piece of scientific research with data that supports the conclusions. Experiments must have been conducted rigorously, with appropriate controls, replication, and sample sizes. The conclusions must be drawn appropriately based on the data presented.

Reviewer #1: Yes

Reviewer #2: Yes

2. Has the statistical analysis been performed appropriately and rigorously?

Reviewer #1: Yes

Reviewer #2: Yes

3. Have the authors made all data underlying the findings in their manuscript fully available?

The [PLOS Data policy](http://www.plosone.org/static/policies.action#sharing) requires authors to make all data underlying the findings described in their manuscript fully available without restriction, with rare exception (please refer to the Data Availability Statement in the manuscript PDF file). The data should be provided as part of the manuscript or its supporting information, or deposited to a public repository. For example, in addition to summary statistics, the data points behind means, medians and variance measures should be available. If there are restrictions on publicly sharing data—e.g. participant privacy or use of data from a third party—those must be specified.

Reviewer #1: Yes

Reviewer #2: Yes

4. Is the manuscript presented in an intelligible fashion and written in standard English?

PLOS ONE does not copyedit accepted manuscripts, so the language in submitted articles must be clear, correct, and unambiguous. Any typographical or grammatical errors should be corrected at revision, so please note any specific errors here.

Reviewer #1: Yes

Reviewer #2: No

5. Review Comments to the Author

Please use the space provided to explain your answers to the questions above. You may also include additional comments for the author, including concerns about dual publication, research ethics, or publication ethics. (Please upload your review as an attachment if it exceeds 20,000 characters)

Reviewer #1: It is an excellent manuscript about a very important surgical issue of training. I commend you for writing it in a very elaborate way describing all the requisite details. The results are in line as evident from published research in simulation training. The language is well-written.

**Response:** We are deeply honored to be highly evaluated from the reviewer 1.

Reviewer #2:

**Reviewer’s comment:** A very interesting study that supports what has been largely vehiculated in literature, the fact that high-volume centers in the case of PD have better results than small volume centers. It is important to keep in mind and the authors should mention this in the introduction that although the surgeon is one of the determining factors of success but he is not the only one. Caring for a patient with PD usually involves a complex team and although the skills of the surgeon are improved the rest of the team must evolve in the same pace.

**Response:** According the reviewer’s comment, we added the following description in the revised manuscript:

Page 4, line 84-89 (new):

Overall, consistently reported relationship between hospital-volume and operative outcome indicated that the care of patients undergoing PD involves variable procedures conducted by a complex team and a role that surgeons’ proficiency in the operation room plays may be merely a part of them. In the present study, we specifically focused on the aspect of surgeon’s role and investigated the feasibility of simulation training of PJ using an inanimate biotissue model.

A few notes:
**Reviewer’s comment:** It is an observational study, the results need to be interpreted in this context with care.

**Response:** According to the reviewer’s comment, we have revised the manuscript as follows:

Page 13, line 288-289 (new):

Finally, this is an observational study and the results need to be interpreted in this context with care.

**Reviewer’s comment:** The authors used the OSATS scale to measure the surgeon evolution. Is there a particular reasons for this scale as there are multiple other options to evaluate surgeon skill.

**Response:** Although there are various measurement tools for surgical skills as the reviewer commented, a review article by van Hove et al. stated that OSATS is presently accepted as the ‘gold standard’ for objective skill assessment (Br J Surg, 2010). Therefore, we chose OSATS for the measurement of surgeon evolution in the present study. We revised the manuscript of Introduction section as follows:

Page 4, line 77-79 (new):

The Objective Structured Assessment of Technical Skills (OSATS) is widely accepted as a reliable objective method for assessing the surgical skill levels of surgeons, and considered as the gold standard for objective skill assessment^19^

**Reviewer’s comment:** Line 150-153 since the study did not require patients I do not think this ethics consent is mandatory.

**Response:** We appreciate the reviewer’s comment, however, to register this study for the Japanese clinical trial registry as the proof of prospective study, the approval of the institutional review board was mandatory.

**Reviewer’s comment:** Line 169 the authors mention a large interval of SFs which were 6 to 17 years from their graduation. Also there is a large variety in number of PD each of them performed before this study which varied from 3 PD to 19 PD. Why were the participants included with such a large variety in years of experience and numbers of PD’s. I think this issue needs to be addressed as it can influence the results in a such a small study.

**Response:** All fellows affiliated with the Department of Hepatobiliary-Pancreatic Surgery, Juntendo University Hospital as of September 2019 participated in the present study. This resulted in a large variety in years of experience and numbers of PDs. According to the reviewer’s comment, we have revised the manuscript as follows:

Page 13, line 291, Page 14, line 292-294 (new):

In addition, there was a large variety in the participants’ demographics with respect to years from their graduation (6 to 17 years) and number of PD they had performed (3 to 19 PDs). These could make the results of this study difficult to be interpreted and could have resulted in selection bias,

6. PLOS authors have the option to publish the peer review history of their article ([what does this mean?](https://journals.plos.org/plosone/s/editorial-and-peer-review-process#loc-peer-review-history)). If published, this will include your full peer review and any attached files.


If you choose “no”, your identity will remain anonymous but your review may still be made public.


**Do you want your identity to be public for this peer review?** For information about this choice, including consent withdrawal, please see our [Privacy Policy](https://www.plos.org/privacy-policy).

Reviewer #1: **Yes:** Mohammed Amir

Reviewer #2: No

[NOTE: If reviewer comments were submitted as an attachment file, they will be attached to this email and accessible via the submission site. Please log into your account, locate the manuscript record, and check for the action link "View Attachments". If this link does not appear, there are no attachment files.]

While revising your submission, please upload your figure files to the Preflight Analysis and Conversion Engine (PACE) digital diagnostic tool, <https://pacev2.apexcovantage.com/>. PACE helps ensure that figures meet PLOS requirements. To use PACE, you must first register as a user. Registration is free. Then, login and navigate to the UPLOAD tab, where you will find detailed instructions on how to use the tool. If you encounter any issues or have any questions when using PACE, please email PLOS at [figures@plos.org](mailto:figures@plos.org). Please note that Supporting Information files do not need this step.
